# Uses and importance of wild fungi: traditional knowledge from the Tshopo province in the Democratic Republic of the Congo

**DOI:** 10.1186/s13002-017-0203-6

**Published:** 2018-02-12

**Authors:** Héritier Milenge Kamalebo, Hippolyte Nshimba Seya Wa Malale, Cephas Masumbuko Ndabaga, Jérôme Degreef, André De Kesel

**Affiliations:** 1 Faculté des Sciences, Université de Kisangani, BP 2012, Kisangani, Democratic Republic of the Congo; 2 Faculté des Sciences, Université Officielle de Bukavu, BP 570, Bukavu, Democratic Republic of the Congo; 3 Botanic Garden Meise, B-1860 Meise, Belgium; 4 Fédération Wallonie-Bruxelles, Service Général de l’Enseignement Supérieur et de la Recherche Scientifique, B-1080 Brussels, Belgium; 5 Centre de Recherches Universitaires du Kivu (CERUKI-ISP), BP 854, Bukavu, Democratic Republic of the Congo

**Keywords:** Ethnomycology, Medicinal fungi, Edible fungi, Tshopo, DR Congo

## Abstract

**Background:**

Wild mushrooms constitute an important non-timber forest product that provides diverse substances and services, especially food and income for local communities from many parts of the world. This study presents original ethnomycological documentation from the dense rainforests of the Democratic Republic of the Congo.

**Methods:**

Ethnomycological surveys were made within local communities near the biosphere reserve of Yangambi and the Yoko forest reserve. The interviews involved 160 informants from six different ethnic communities (Bakumu, Turumbu, Topoke, Lokele, Ngelema, and Ngando). Specific reported use (RU), the relative importance (RI), and the cultural significance (CS) of wild edible fungi were calculated using quantitative data from enquiries.

**Results:**

The people from Tshopo use 73 species of wild mushrooms either for food (68 species), as medicine (9 species), in a recreational context (2 species), or related to myths and beliefs (7 species). Women are more involved in harvesting and are the main holders of cultural aspects related to fungi. The results show that knowledge of useful mushrooms differs between ethnic groups. The Ngando people have the highest ethnomycological expertise, which is expressed in their extensive cultural and practical use of fungi. *Pleurotus tuber-regium* is the most important species (MCSI = 1.9 and *p* value < 2.2e^−16^) as it is being used for food, as a medicine, and more. *Daldinia eschscholtzii* is the most important (MUI = 0.86 and *p* value < 2.2e^−16^) for medicinal applications, while *Schizophyllum commune*, *Auricularia cornea*, *A. delicata*, *Marasmius buzungolo*, and *Lentinus squarrosulus* are mostly appreciated for food. The latter five species are all wood-decaying saprotrophs.

**Conclusion:**

Despite the presence of edible ectomycorrhizal taxa in the dense rainforests of Tshopo, local people only seem to have an interest in saprotrophic taxa. Some mushroom pickers deliberately cut down host trees to promote the development of saprotrophic taxa. Inducing forest degradation is considered beneficial as it promotes the development of saprotrophic taxa. The domestication of locally appreciated saprotrophic lignicolous fungi is proposed as a mitigating measure against fellings.

## Background

In many parts of the world, wild mushrooms constitute important non-timber forest product (NTFP) [1–4]. They provide diverse substances and services to local communities, especially as a source of food and income [4–7]. Nutritionally, they are an important source of proteins, vitamins, fats, carbohydrates, amino acids, and minerals [8, 9], i.e., a worthy alternative or substitute for meat and fish [6, 8]. In various cultures, mushrooms are widely used as medicine, but also in a recreational context and for myths and beliefs [10].

Several studies note that local mushroom knowledge varies with people’s cultures and beliefs [10–13]. Within local communities, the traditional knowledge is passed on from one generation to another. For local communities, it is often the only, albeit fragile but effective, way of safeguarding information concerning useful fungi [11–13]. Traditional knowledge linked to fungal taxa can be recorded and allows to evaluate its use-value for a specific local community [11, 14]. The analysis of use-value provides information about cultural differences between communities [11, 14].

Scientists recognize that assessing the various uses of wild fungi by local people is the key towards a better valorization of services provided by wild useful fungi [7]. This also enables to better elaborate participative management and conservation plans [15]. Several scientists [15] have reported that pressure caused by mushrooms pickers, either deliberate by fellings or non-deliberate by trampling, induces forest degradation. Forest conservation strategies aiming at a sustainable mushroom harvest largely depend on the targeted fungal ecological group (saprotrophic and/or ectomycorrhizal). While ectomycorrhizal taxa constitute the most important group of useful fungi in miombo woodlands and open forests [16, 17], the saprotrophic taxa seem more important in rain and montane forest types [3, 18, 19]. Cultivation of locally used fungi is considered as a mitigating measure against deliberate fellings for the promotion of some useful saprotrophic taxa. An ethnomycological survey is thereby important before the cultivation starts, to collect and document about the locally used fungal species.

Documentation related to the traditional use of mushrooms is scarce [3, 10, 12, 13]. Within local communities, oral transmission is often the only way to transfer local knowledge [12, 13]. The lack of documentation regarding wild edible fungi and the need to conserve it through oral transmission makes local mycological knowledge fragile [17]. Due to a continuing exodus of younger people to new areas or the bigger cities, local communities gradually lose an important part of their traditional knowledge, particularly about mushrooms [12, 13, 17]. There is a genuine need to record and document local traditional knowledge about wild edible and useful fungi.

The Tshopo province (Democratic Republic of the Congo) is part of the central African Congo basin and its rainforests are host to a wealth of edible and useful fungal species [3]. In this region, more than 80% of the population lives outside the cities [20, 21] and depend for their livelihoods on wild natural resources, including mushrooms. This study aims to fill the gap in ethnomycological knowledge available from the DR Congo by delivering original data obtained from the communities living around the Man-and-Biosphere Reserve of Yangambi and the Yoko Forest Reserve.

## Methods

### Study site

**Fig. 1:**
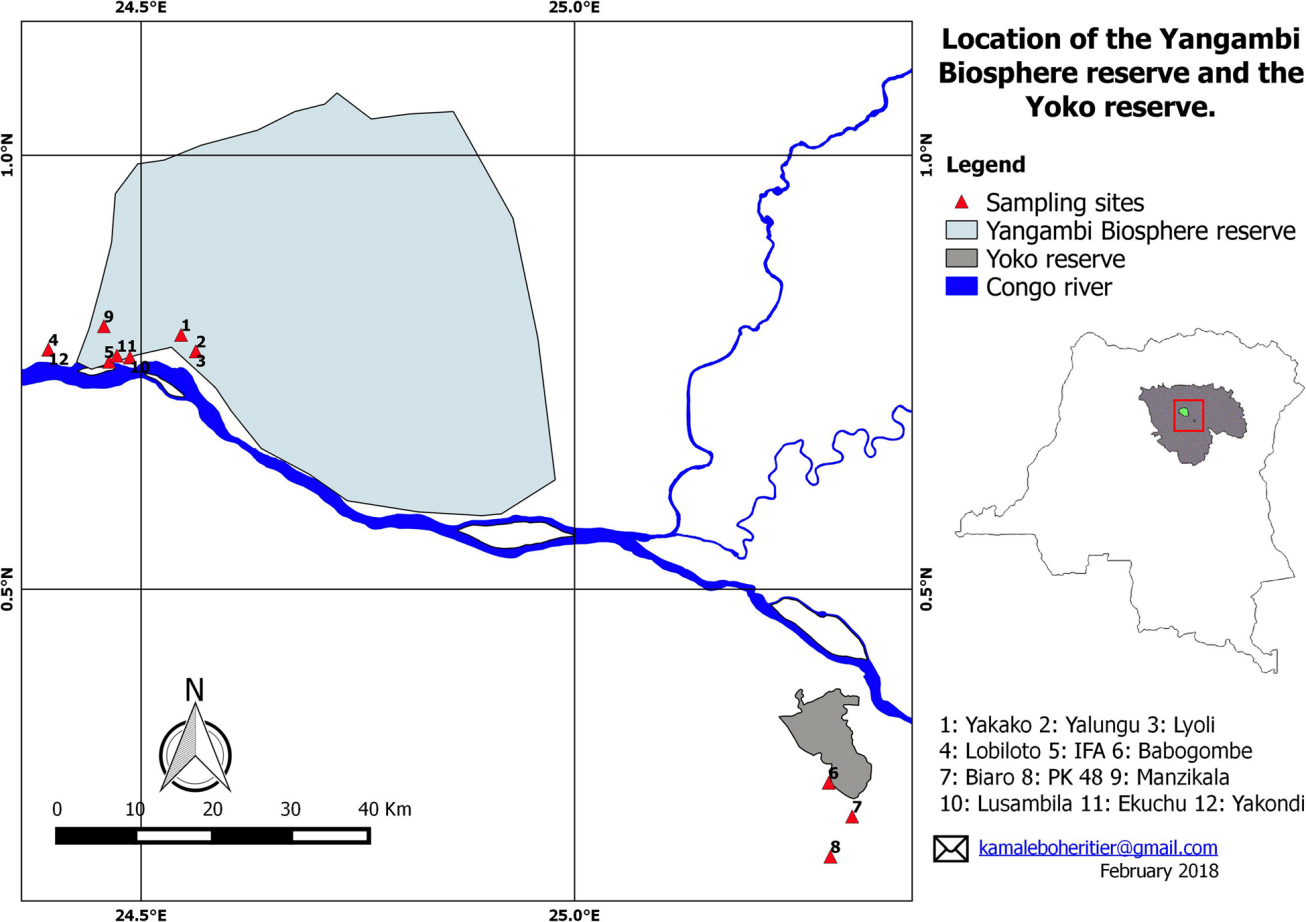
Location of the study site

The study sites are located in the Tshopo province of the Democratic Republic of the Congo. The ethnomycological survey was carried out in villages surrounding the Yangambi Man-and-Biosphere Reserve (0° 51′ 01.62″ N; 24° 31′ 43.53″ E) (Isangi territory) and the Yoko Forest Reserve (0° 17′ 34.9″ N; 25° 18′ 27.4″ E) in Ubundu territory (Fig. 1). The region is mainly characterized by semi-deciduous rainforests dominated by *Gilbertiodendron dewevrei* (De Wild.) J. Léonard, *Scorodophloeus zenkeri* Harms, *Prioria balsamifera* (Vermoesen) Breteler, and *Julbernardia seretii* (De Wild.) Troupin [22–25].

As part of the equatorial region, the Tshopo province has a rainy and hot climate, ranged on the A_f_ type according to the classification of Köppen [26]. The climate is characterized by monthly temperatures ranging between 22.4 and 29.3 °C, with an annual average around 25 °C. The annual rainfall ranges from 1600 to 2200 mm with an average around 1828 mm. Rainfall is irregular throughout the year. The average year typically has a long rainy season interrupted by two small drier seasons, i.e., from December till January and from June till August [22, 26].

### Sampling methods and data collection

Fungal sampling and ethnomycological survey have been carried out during the main rainy season (from March to June) of the years 2015 and 2016. The identification of the useful fungi was done using macro- and micromorphological features given in taxonomical contributions [16, 27–35]. Species names and author’s abbreviations were annotated according to the Index Fungorum site [36]. The dried fruiting bodies are deposited at the herbarium of the Botanic Garden Meise in Belgium (BR).

Ethnomycological data were collected randomly using open and semi-structured interviews. The interview involved mainly the available household head, sometimes assisted by other family members. Face-to-face interviews were preferred over paper fill-in questionnaires as some of the informants preferred to respond to oral questions [3, 12, 19].

The questions focused basically on the informant’s knowledge concerning the different uses of wild fungi. The interview campaign involved 160 informants, all randomly selected from six different ethnic communities (Bakumu, Turumbu, Topoke, Lokele, Ngelema, and Ngando). The interviewed communities belong to four local villages in the vicinity of the Yangambi Man-and-Biosphere Reserve (Yakako, Yalungu, Lyoli, and Lobiloto), four quarters in Yangambi city (IFA, Lusambila, Ekuchu, and Manzikala), and four villages surrounding the Yoko Forest Reserve (Babogombe, Biaro, PK 48, and PK 25).

Referring to the 2014 annual report of people census from the Turumbu sector, and based on a preliminary survey within the study area, the entire study involved about 600 households. The mean distribution was around 25 households per village (4.2% of the entire pool) and 150 (25%), 120 (20%), 80 (13.3%), and 50 (8.3%) households respectively reported from Ekuchu, Lusambila, Manzikala, and IFA. The sample size of interviewed households (*n*) was calculated by the following formula: *n* = $\frac{\mathrm{N}}{1+\mathrm{N}\ast {\mathrm{e}}^2}$ [37] where *N* = the total number of available households and e is the level of precision. Considering a ± 7% precision level, the sample size was calculated as follows: *n* = $\frac{600}{1+\left(N\times {0.07}^2\right)}$≈ 152 households, fitted to 160. Therefore, 7 households were interviewed from each of the 4 villages while 38 households from Ekuchu, 30 from Lusambila, 20 from Manzikala, and 12 from IFA.

### Data analysis

The here analyzed data only refer to locally known useful fungi and allow to present (per species) information on the specific reported use (RU), the relative importance (RI), and the cultural significance (CS). According to [11], such topics have been applied for plants [38–41] and adapted for fungi since 2001 [42]. The specific reported use of a given folk or biological fungal taxon is the number of uses locally assigned to it, and this according to all the informants interviewed [11, 15]. It represents the number of times a use category is reported by the informant from a given species. Modified from several previous studies [14, 42]; the reported use is calculated as follows: RU =∑*U*_*i*_ (where *U*_*i*_ is the specific use assigned by each informant to a given species).

The relative importance is also calculated by the following formula: RI = NUC + NT, where “NUC” is the number of use categories of a given species divided by the total number of use-categories of the most useful species and “NT” is the number of types of uses assigned to the same species divided by the total number of types of uses attributed to the most important species [15]. The value of the relative importance for each species is based on a score scale from 0 to 2.

The cultural significance concerns the ethnic index of cultural significance (EICS) and the mushrooms’ cultural significance index (MCSI). The mushrooms’ cultural significance index refers to the importance of the role that a given fungal taxa plays in social life and behavior of a certain community [42]. EICS gives additional information about cultures and communities to whom mushrooms are more important and useful, and that have much mushroom knowledge to be shared and transmitted to future generations [11, 15, 42]. As four categories for use were reported (all species), the cultural significance score (CS) ranges from 1 to 4.

The calculation of cultural significance index is borrowed from models developed by numerous scientists [11, 42] and modified according to the present study context. It is obtained by summing of the cultural significance sub-indexes for each use category and species. The cultural significance calculation included the index of frequency of mention fitness (Q*i*), the mushrooms’ edibility index (MEI), medicinal use index (MUI), recreational use index (RUI), and belief and myth use index (BUI). All sub-indexes were attributed the same weight and are based on a binary score from 0 to 1. The taste appreciation index (TAI) was assessed to list the tastiest and most appreciated edible fungi. The index of frequency of mention fitness is obtained by dividing 1 by the total number of species reported for a given use category (for EICS) or by the total number of informants (for MCSI). Q*i* = $\frac{1}{Ni}$, where N*i* = total number of species reported for the use or total number of informants. All sub-index scores were assigned through informants’ answers to the following questions:

- MEI: Do you eat this mushroom? (Yes = 1, No = 0)
- TAI: Is it your most preferred and appreciated mushroom? (Yes = 1, No = 0)
- MUI: Is this mushroom used as a medicine? (Yes = 1, No = 0)
- What other uses do you know for this mushroom? (recreational, belief, and myth):
- Recreational use (RUI): Yes = 1, No = 0,
- Belief or mythical use (BUI): Yes = 1, No = 0

Finally, the cultural significance index (CSI) was calculated by the following formula:

CSI = $$ \left[{\sum}_i^n\left(\mathrm{MEI}\right)i+{\sum}_i^n\left(\mathrm{MUI}\right)i+{\sum}_i^n\left(\mathrm{RUI}\right)i+{\sum}_i^n\left(\mathrm{BUI}\right)i\right]{Q}_{i.} $$

Statistical tests were conducted in R software. The difference between eaten species number by different ethnic groups were analyzed by Tukey HSD multiple comparison test. The chi-squared test was performed to assess the difference between the numbers of reported fungi species for each use categories and between the different fungal functional group. In addition, the correspondence analysis (CA) performed by FactoMineR package was used to map the most important edible fungi within the tribes. The cluster analysis in Past software was also used to assess the similarity of mushrooms’ consumption between people.

## Results

In total, 160 informants (72 women, 88 men) were interviewed. The age of the informants ranged from 16 to 72 years. From the entire sample, the Turumbu people were the most dominant tribe with 67 informants (41.9%), while the rest were Topoke (17.5%), Lokele (15%), Ngelema (10%), Bakumu (8.8%), and Ngando (6.9%). The entire pool of informants recognized 73 species of wild fungi as useful (Table 1).

**Table 1:** List of useful fungi and the values of their cultural significance index (RI = relative importance; MUI = medicinal use’s index, MCSI = mushroom cultural significance index)

Family	Species	Voucher number	Reported uses	RI	MUI	MCSI
*Agaricaceae*	*Agaricus bambusicola* Heinem.	ADK5267	Food	0.4	0.00	0.15
	*Agaricus crocopeplus* Berk. & Broome	ADK5747, MKH073	Food	0.4	0.00	0.17
	*Cystolepiota* sp.	ADK5277	Food	0.4	0.00	0.24
	*Hymenagaricus luteolosporus* Heinem. & Little Flower	ADK5222	Food	0.4	0.00	0.11
	*Leucocoprinus cepistipes* (Sowerby) Pat.	ADK5206	Food	0.4	0.00	0.12
*Amanitaceae*	*Amanita annulatovaginata* Beeli	MKH016	Food	0.4	0.00	0.26
	*Amanita echinulata* Beeli	ADK5938, MKH159	Food	0.4	0.00	0.22
	*Amanita marmorata* Cleland & E.-J. Gilbert	ADK5903	Food	0.4	0.00	0.21
	*Amanita pudica* (Beeli) Walleyn	ADK5924	Food	0.4	0.00	0.29
	*Amanita robusta* Beeli	ADK5236	Food	0.4	0.00	0.31
*Auriculariaceae*	*Auricularia cornea* Ehrenb.	ADK5175	Food	0.4	0.00	1.07
	*Auricularia delicata* (Mont.ex Fr.) Henn.	ADK5169, MKH214	Food, medicinal	0.8	0.05	1.08
*Boletaceae*	*Rubinoboletus luteopurpureus* (Beeli) Heinem. & Rammeloo	ADK5192, MKH235	Food	0.4	0.00	0.15
*Cantharellaceae*	*Cantharellus congolensis* Beeli	ADK5199, MKH180	Food	0.4	0.00	0.56
	*Cantharellus densifolius* Heinem.	MKH017	Food, myth and belief	0.8	0.00	0.38
	*Cantharellus floridulus* Heinem.	ADK5670	Food	0.4	0.00	0.34
	*Cantharellus isabellinus* Heinem.	ADK5667	Food	0.4	0.00	0.39
	*Cantharellus longisporus* Heinem.	ADK5684, MKH141	Food	0.4	0.00	0.87
	*Cantharellus luteopunctatus* (Beeli) Heinem.	ADK5836, MKH078	Food	0.4	0.00	0.32
	*Cantharellus miniatescens* Heinem.	ADK5216	Food	0.4	0.00	0.24
	*Cantharellus pseudofriesii* Heinem.	ADK5232	Food	0.4	0.00	0.21
	*Cantharellus ruber* Heinem.	ADK5749	Food	0.4	0.00	0.18
	*Cantharellus rufopunctatus* (Beeli) Heinem.	ADK5892, MKH164	Food	0.4	0.00	0.53
*Ganodermataceae*	*Ganoderma* sp.	ADK5854	Medicinal use	0.6	0.51	0.51
*Hygrophoraceae*	*Hygrocybe cantharellus* (Schwein.) Murrill	ADK5849, MKH229	Food	0.4	0.00	0.18
*Lyophyllaceae*	*Termitomyces mammiformis* Heim	MKH126	Food	0.4	0.00	0.41
	*Termitomyces robustus* (Beeli) Heim	ADK5242	Food, myth and belief	0.8	0.00	0.98
	*Termitomyces* sp.1	ADK5852	Food	0.4	0.00	0.65
	*Termitomyces* sp.2	ADK5179	Food	0.4	0.00	0.78
*Marasmiaceae*	*Calyptella longipes* (Cooke & Massee) W.B. Cooke	ADK5868	Food	0.4	0.00	0.08
	*Gymnopus* sp.	ADK5884	Food	0.4	0.00	0.45
	*Marasmius arborescens* (Henn.) Beeli	ADK5255, MKH231	Food	0.4	0.00	0.62
	*Marasmius bekolacongoli* Beeli	ADK5779, MKH035	Food	0.4	0.00	0.39
	*Marasmius buzungolo* Singer	ADK5760, MKH121	Food	0.4	0.00	1.04
	*Marasmius confertus* Berk. & Broome	ADK5276, MKH021	Food	0.4	0.00	0.32
	*Marasmius* sp.	MKH226	Food	0.4	0.00	0.14
	*Trogia infundibuliformis* Berk. & Br.	ADK5223	Food	0.4	0.00	0.24
*Mycenaceae*	*Mycenoporella* sp.	ADK5665, MKH084	Food	0.4	0.00	0.11
*Phallaceae*	*Phallus indusiatus* Vent.	ADK5822	Myth and belief	0.4	0.00	0.04
*Physalacriaceae*	*Armillaria heimii* Pegler	ADK5230, MKH046	Food	0.4	0.00	0.49
*Pleurotaceae*	*Pleurotus cystidiosus* O.K. Mill.	ADK5272, MKH067	Food	0.4	0.00	0.79
	*Pleurotus flabellatus* (Berk. & Br.) Sacc.	ADK5771	Food	0.4	0.00	0.30
	*Pleurotus tuber-regium* (Fr.) Fr.	ADK5183, MKH106	Food, medicinal, myth and belief, and recreational use	2.0	0.57	1.90
*Pluteaceae*	*Volvariella* sp.	ADK5786	Food	0.4	0.00	0.26
*Polyporaceae*	*Lentinus brunneofloccosus* Pegler	MKH048	Food	0.4	0.00	0.21
	*Lentinus squarrosulus* Mont.	ADK5226, MKH079	Food, myth and belief	0.8	0.00	1.00
	*Lentinus velutinus* Fr.	ADK5262, MKH194	Medicinal use	0.4	0.08	0.08
	*Favolus tenuiculus* (P. Beauv.) Fr.	MKH109	Food	0.4	0.00	0.11
	*Pycnoporus sanguineus* (L.) Murrill	ADK5873, MKH080	Medicinal use	0.4	0.76	0.76
*Psathyrellaceae*	*Coprinellus disseminatus* (Pers.) J.E. Lange	ADK5615	Food	0.4	0.00	0.11
*Repetobasidiaceae*	*Cotylidia aurantiaca* (Pat.) A. L. Welden	ADK5625, MKH058	Food	0.4	0.00	0.19
*Russulaceae*	*Lactarius acutus* R. Heim	ADK5251, MKH001	Food	0.4	0.00	0.31
	*Lactifluus annulatoangustifolius* (Beeli) Buyck	ADK5862	Food	0.4	0.00	0.18
	*Lactifluus pelliculatus* (Beeli) Buyck	MKH012	Food	0.4	0.00	0.21
	*Lactarius* sp.	MKH075	Food	0.4	0.00	0.16
	*Russula annulata* R. Heim	ADK5243, MKH014	Food	0.4	0.00	0.20
	*Russula inflata* Buyck	ADK5217	Food	0.4	0.00	0.18
	*Russula meleagris* Buyck	ADK5708, MKH113	Food	0.4	0.00	0.22
	*Russula porphyrocephala* Buyck	ADK5750, MKH178	Food	0.4	0.00	0.16
	*Russula pruinata* Buyck	ADK5193, MKH115	Food	0.4	0.00	0.17
	*Russula striatoviridis* Buyck	ADK5896	Food	0.4	0.00	0.33
	*Russula sesemoindu* Beeli	ADK5816, MKH146	Food, myth and belief	0.8	0.00	0.38
*Sarcoscyphaceae*	*Cookeina speciosa* (Fr.) Dennis	ADK5171, MKH054	Food, recreational use	1.3	0.00	0.53
*Schizophyllaceae*	*Schizophyllum commune* Fr.	MKH108	Food, medicinal use, myth and belief	1.4	0.13	1.45
*Strophariaceae*	*Gymnopilus zenkeri* (Henn.) Singer	ADK5619	Food	0.4	0.00	0.52
*Tapinellaceae*	*Tapinella panuoides* (Fr.) E.-J. Gilbert	MKH043	Food	0.4	0.00	0.16
*Tricholomataceae*	*Clitocybula* sp.	ADK5220	Food	0.4	0.00	0.26
	*Collybia aurea* (Beeli) Pegler	ADK5211	Food	0.4	0.00	0.40
	*Collybia* sp.	MKH154	Food	0.4	0.00	0.36
	*Lepista* sp.	MKH085	Food	0.4	0.00	0.34
	*Leucopaxillus* sp.	ADK5229	Food	0.4	0.00	0.16
*Xerocomaceae*	*Xerocomus spinulosus* Heinem. & Gooss.-Font.	ADK5209	Food	0.4	0.00	0.08
*Xylariaceae*	*Daldinia eschscholtzii* (Ehrenb.) Rehm	ADK524, MKH179	Medicinal use	0.6	0.86	0.86
	Chi squared test	*p* value		0.00598	2.2e^−16^	2.2e^−16^
		Significance		**	***	***

***Very high significant difference; **High significant difference

**Fig. 2:**
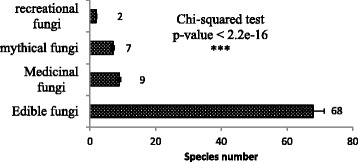
Number of fungal species by use category

Four categories of usage were reported (Fig. 2): food, medicinal, belief and myth, and recreational.

**Fig. 3:**
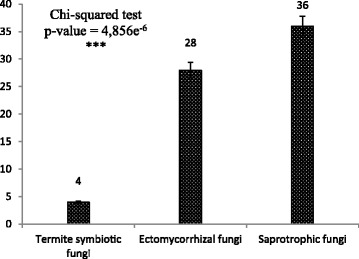
Distribution of edible fungi within functional groups

Among the 73 fungal species reported as useful, 68 are mentioned as edible, only 9 are reported as medicinal, 7 are reported as mythical, and 2 are used in a recreational context. Some edible taxa such as *Schizophyllum commune* and *Pleurotus tuber-regium* were used both for food and medicine. Figure 3 shows the distribution of edible fungi within functional groups.

The reported edible species belong to 3 fungal functional groups. The most commonly eaten and appreciated species belong to the saprotrophs (36 species), most of these within the *Marasmiaceae.* Among ectomycorrhizal fungi, the *Cantharellaceae* and *Russulaceae* were the most represented (11 species each).

### Mushrooms’ edibility and taste appreciation

**Fig. 4:**
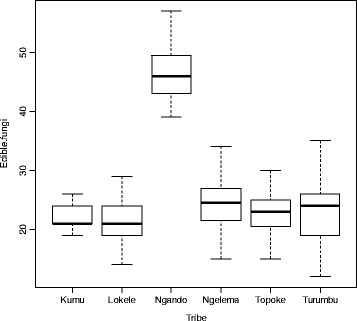
Boxplots showing the distribution patterns of number of fungi consumed by different tribes

Qualitatively, mushroom consumption clearly differs from one ethnic group to another (Fig. 4). Ngando people use more than 50 fungal species for food. They are followed by Turumbu and Ngelema while both Kumu and Ngelema consume only a restricted number of species. The number of consumed fungi among the Turumbu people is very variable. Some of them eat less than 15 species while others eat more than 35 species.

**Fig. 5:**
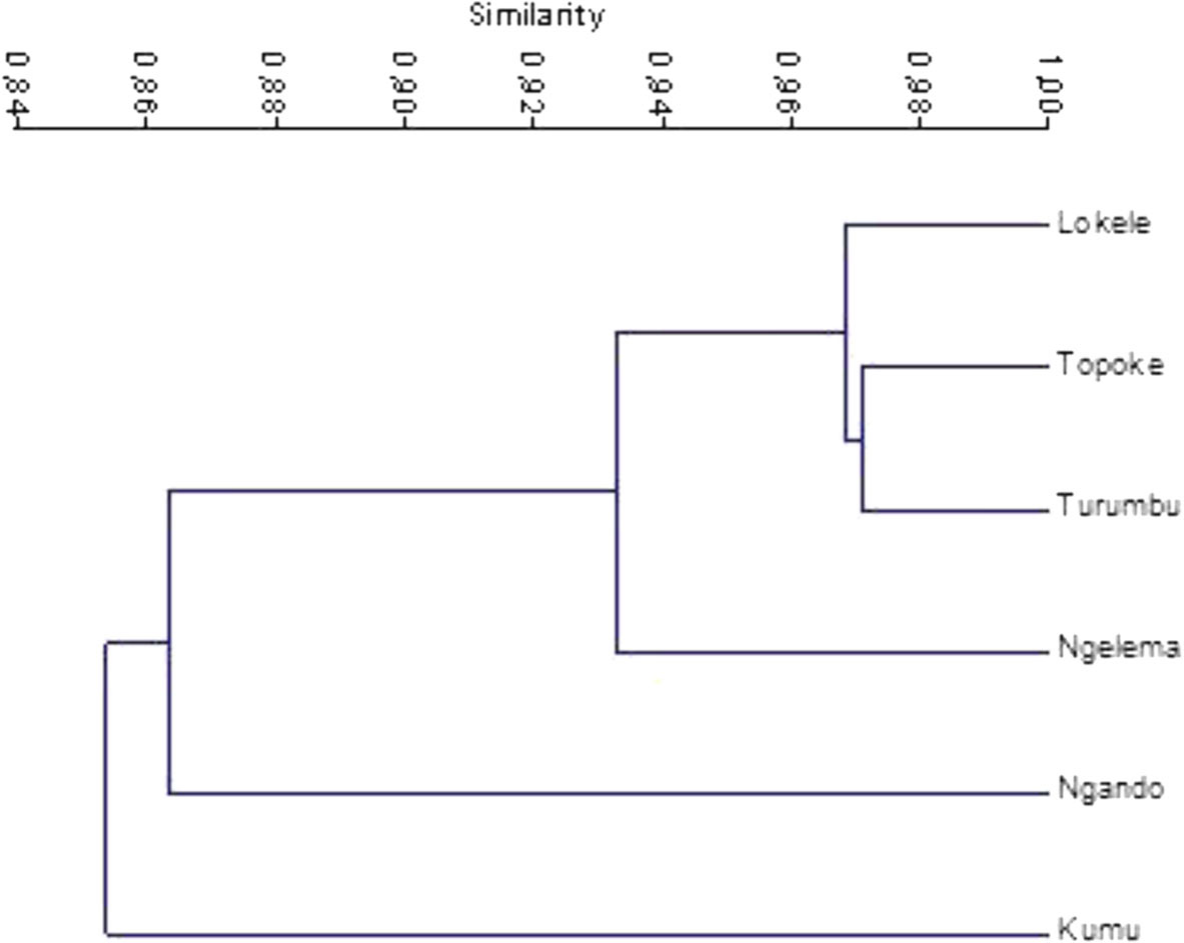
Cluster diagram showing the similarity of fungi diets chosen by the studied tribes

The Tukey HSD multiple comparison test shows a highly significant difference between the number of edible mushrooms consumed by Ngando and other ethnic groups (*p* value < 0.001). Except for the Ngando people, who consume more species, we observe that the number of consumed taxa is not significantly different among all the other ethnic groups. The level of similarity of edible fungi used for consumption is presented in Fig. 5.

**Fig. 6:**
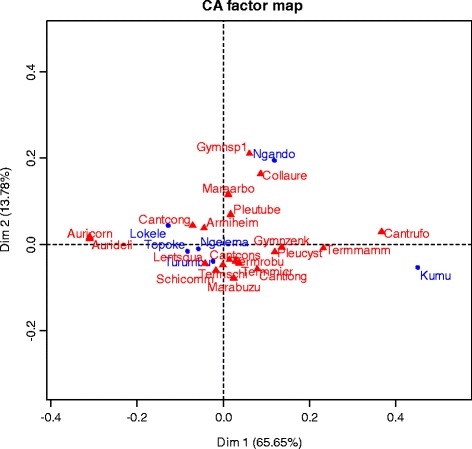
Two main axes of the correspondence analysis (CA) of the most eaten fungi. Legend: red: fungi names, Blue: ethnic groups. The two axes explain 79.43% of ordination of edible fungi within ethnic groups (65.65% for the first axis and 13.78% for the second axis)

A cluster analysis (Fig. 5) shows that Topoke, Lokele, and Turumbu use a very similar set of species (more than 96% of similarity) for consumption. The Ngando and Kumu are isolated and consume other mushroom species, clearly not eaten by the other groups. The fungal diet of the Ngelema people seems intermediate (Fig. 5). The ordination map (Fig. 6) of the 20 most consumed species (with highest edibility index) shows the variation of mushroom consumption within different ethnic groups.

Some ethnic groups consume specific fungi, clearly separating their mushroom diet or consumption profile from the others. *Cantharellus rufopunctatus* and *Termitomyces mammiformis* for example constitute the most eaten and distinctive fungal species used by the Kumu people. *Gymnopus* sp., *Collybia aurea*, *Marasmius arborescens*, and *Pleurotus tuber-regium* are particularly eaten by Ngando people. *Auricularia cornea* and *A. delicata* are mostly eaten by Lokele, Topoke, and Turumbu, while all the preferred species from the Ngelema people are shared by all ethnic groups. The Ngelema people do not seem to have a particular mushroom diet. In addition, the correspondence analysis shows that *Lentinus squarrosulus*, *Schizophyllum commune*, *Marasmius buzungolo*, *Termitomyces* sp., *Cantharellus longisporus*, and *Termitomyces robustus* are eaten by all the ethnic groups.

From the entire pool of informants, the most commonly eaten mushroom is *Schizophyllum commune*. It is followed by *Lentinus squarrosulus*, *Auricularia cornea* and *A. delicata*, *Marasmius buzungolo*, *Cantharellus longisporus*, *Termitomyces robustus*, *Termitomyces microcarpus*, *Pleurotus cystidiosus*, and *Termitomyces* sp. Nevertheless, *Auricularia* and *Russul*a are refused by Kumu people. In their native language, these species are called “Sengwa”, which means inedible.

The taste-based appreciation varies with each ethnic group. From all ethnic groups, the 10 most tasty and appreciated species were listed. In all tested ethnic groups *Schizophyllum commune*, *Lentinus squarrosulus*, and *Marasmius buzungolo* were reported as the tastiest and most appreciated taxa. However, some Kumu interviewees reported *Pleurotus cystidiosus*, *Termitomyces robustus*, and *Cantharellus longisporus* tastiest. *Auricularia* species were appreciated and reported tastiest by some Topoke, Lokele, Turumbu, and Ngelema interviewees.

### Medicinal fungi and their applications

**Fig. 7:**
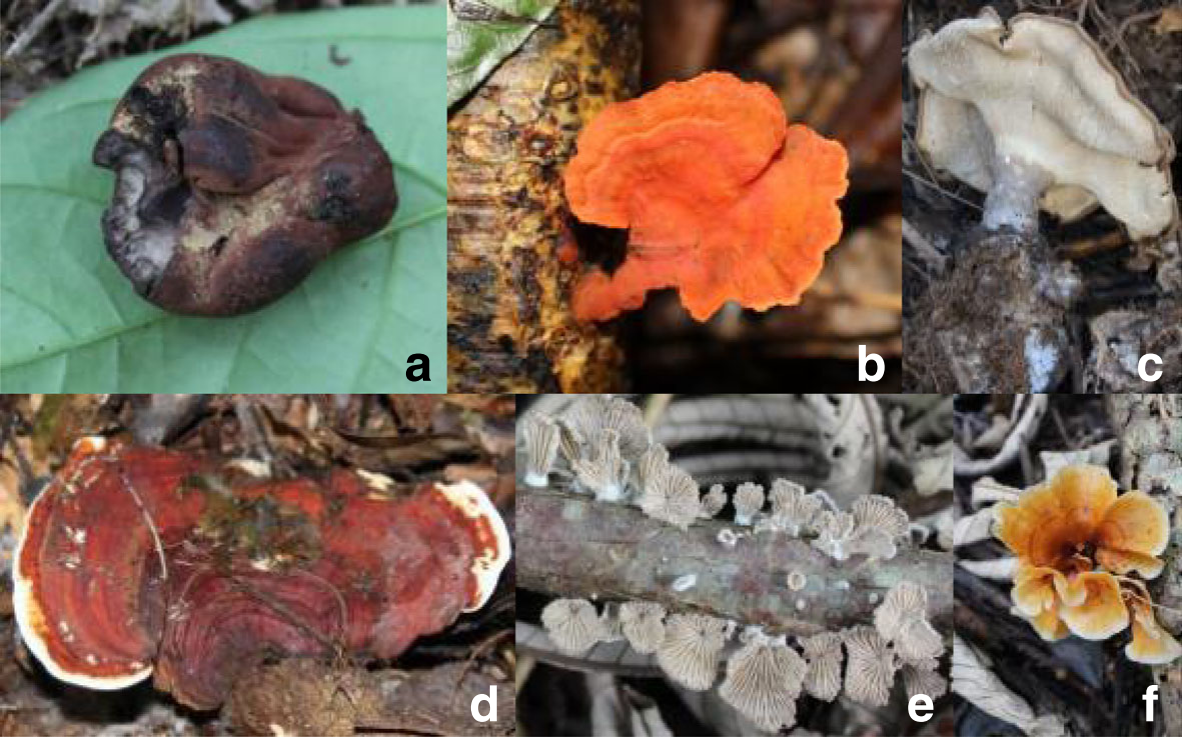
The sporocarps of the most used medicinal fungi. **a** *Daldinia eschscholtzii*. **b** *Pycnoporus sanguineus*. **c** *Pleurotus tuber-regium*. **d** *Ganoderma* sp. **e** *Schizophyllum commune*. **f** *Cotylidia aurantiaca*

Nine fungal species were reported as medicinal. Each species treats specific diseases, although some of them are used to treat several sores and illnesses. *Daldinia eschscholtzii* is the best known medicinal fungus, i.e., used by more than 80% of all the informants. It is used to treat spleen illness and wounds. *Pycnoporus sanguineus* is the next best medicinal fungus and used to treat otitis. The same use against otitis is also reported for *Cookeina speciosa*. Furthermore, *Pleurotus tuber-regium* is used to treat bronchitis, but also for breastmilk stimulation and against bed watering. *Ganoderma* sp*.* and *Lentinus velutinus* are used to treat the male sexual impotence. Moreover, *Schizophyllum commune* is used to treat wounds and breast inflammation. *Auricularia delicata* is used against furonculosis and skin inflammations. *Cotylidia aurantiaca* is used to treat conjunctivitis. Figure 7 presents the photographs of commonly used medicinal fungi.

### Mythical and recreational fungi

Beliefs and myths about mushrooms constitute part of the rich culture of many people from Tshopo province. The most common mythical uses are those meant to bring luck (*Cantharellus densifolius*, *Lentinus squarrosulus*), or to cause discord between friends and lovers (*Russula sesemoindu* and *Phallus indusiatus*). It is also a custom to use powder from *Pleurotus tuber-regium* to chase away birds from rice fields. Out of fear of becoming orphans, children are forbidden to eat *Termitomyces robustus*, and young mothers suffering *Trichomonas vaginalis* are forbidden to eat *Schizophyllum commune* in fear of crib death.

Only two species were reported having some recreational purpose. Nineteen (19%) of the informants state that *Pleurotus tuber-regium* is used by children for recreation. The sclerotium is cut and used to make tires of toy cars. *Cookeina speciosa* is also used by children. The cup-shaped sporophores are collected and thrown to each other for amusement. This particular type use of *Cookeina speciosa* is known by almost 40% of the informants.

### Cultural significance and relative importance

**Fig. 8:**
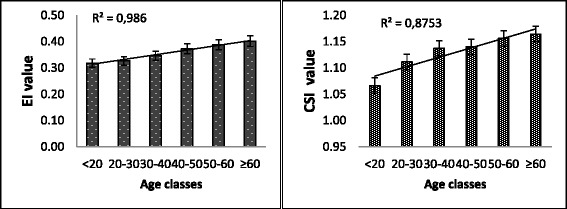
Variation of the cultural significance and knowledge on edible fungi by age. Legend: EI = edibility index, CSI = cultural significance index

Women informants seem to possess most of the knowledge about mushrooms. They possess lots of knowledge about edible fungi and have high value of ethnic index of cultural significance on mushrooms. Although women have a lot of knowledge on mushrooms, the cultural significance remains unequally shared between the ethnic groups. From all ethnic groups interviewed, the Ngando people possess important knowledge on edible fungi as well as wider cultural significance. Ngelema possess more cultural significance than edibility value. The Lokele is still the ethnic group with less cultural significance while the Kumu, Topoke, and Turumbu have significant knowledge compared to Lokele. Furthermore, the consumption and cultural significance on mushrooms seems to increase with age. Young people are mostly involved in picking the more common edible fungi. There is a clear learning curve as old people provide high cultural significance and know more edible taxa than young people (Fig. 8).

According to scores assigned to all species from all used categories, *Pleurotus tuber-regium* is the most culturally significant and important species for local people in Tshopo province. The species is used by a substantial amount of informants, i.e., as food, as medicine, for myth and belief, and in a recreational context. From all investigated taxa, it also happens to be the species producing the largest and the heaviest sporophores. In terms of cultural significance, *Pleurotus tuber-regium* is respectively followed by *Schizophyllum commune*, *Lentinus squarrosulus*, *Auricularia delicata*, *A. cornea*, and *Marasmius buzungolo*.

## Discussion

The study shows that local people from Tshopo, regardless of their age, use wild mushrooms either for food, for medicine, for recreation, or in relation to certain beliefs or myths. This result concurs with many other studies [1, 13, 43]. The study also reports that mushroom knowledge is differently shared within the ethnic groups, as well as age and gender classes. Younger informants know only common edible taxa and a few species useful to make toys with. The older generations, however, handle more species and are skilled with medicinal and mythical uses of fungi. Women are generally more involved in mushroom harvesting and possess very high knowledge on edible taxa as well as their wider cultural significance. This observation is in line with the findings of several other authors [10, 12, 17]. They respectively found that in rural areas in Benin, Zambia, and Tanzania, women and children constitute the most important group of mushroom pickers and sellers. In West Africa, DSRP and PNUD [19, 43] found that basic ethnomycological knowledge crosses gender, age groups, and occupation categories.

Several other studies [1, 12, 13] have shown that people lose part of their mycological knowledge when moving to a new area. However, the Ngando people who came from the former province of Equateur still provide a lot of information about mushrooms, including cultural significance. The high cultural significance of Ngando people on mushroom uses may be explained by the fact that Ngando people are a nearly autarkic community, i.e., living remotely in forest areas and mainly using natural resources for their subsistence. This comment is supported by numerous scientists [44] who reported that Ngando people use mainly wild useful plants for subsistence because of their localization far inside forests. Because of taboos, e.g., not eating any *Auricularia* and *Russula* species (“Sengwa” = inedible fungi), the Kumu people use far less mushrooms. Taboos on mushrooms consolidate mycophobia and negatively affect the people’s interest in mushroom consumption.

Wood-decomposing fungi such as *Schizophyllum commune*, *Auricularia* spp., *Marasmius buzungolo*, and *Lentinus squarrosulus* are the most eaten taxa in the Tshopo province. Several scientists [3, 6, 19, 43] have mentioned a higher consumption interest for saprotrophic species in rainforests. In central African rainforests for example, the saprotrophic fungi constitute the most consumed group [3]. According to several other ethnomycological studies [7, 9, 16, 17, 19, 45], people from miombo forests and savannah woodlands eat significantly more ectomycorrhizal fungi than saprotrophic ones. The main reason for this is that these forests are prone to bushfires that significantly reduce the amount of substrate available for saprotrophs to develop.

Concerning the medicinal use, we report nine species (*Auricularia delicata*, *Cookeina speciosa*, *Daldinia eschscholtzii*, *Cotylidia aurantiaca*, *Ganoderma* sp., *Pleurotus tuber-regium*, *Lentinus velutinus*, *Pycnoporus sanguineus*, and *Schizophyllum commune*) possibly providing medicinal contents. Several studies [1, 46] reported that *Ganoderma* species are the most valuable medicinal fungi worldwide. Even in modern medicine, these taxa are considered genuinely interesting [46]. The reported medicinal use of *Pycnoporus sanguineus*, i.e., to treat ear inflammation, corresponds with the findings of numerous scientists [47] who found that *P*. *sanguineus* is used to treat sores in Malaysia. Moreover, several other scientists [47–49] have reported the medicinal property of *Pleurotus tuber-regium*. According to this study, *Pleurotus tuber-regium* is also used to stop children from bed watering while in Malaysia local people use *Xylaria polymorpha* for this purpose [47].

In addition, the findings report several mythical and belief uses of mushrooms from Tshopo. *Pleurotus tuber-regium* is used to drive away destructive birds from rice fields. *Phallus indusiatus* is used to induce discord between people while *Cantharellus densifolius* and *Lentinus squarrosulus* bring luck. The mythical use of wild fungi or plants has been reported by several studies [40, 43, 49, 50]. From Yoruba people in western Nigeria, *Pleurotus tuber-regium* is considered as the crops of divine gods and used to overpower evil spirits [49]. In Madagascar, the species is used against strong wind, and used by women to get in funerary room without fear of death [49]. These reports of mythical uses might confirm the hallucinogenic effects of some mushrooms mentioned by several previous studies [43, 49, 50].

## Conclusion

*Pleurotus tuber-regium* is reported the most useful and important fungus as it plays a key role in social life and culture of people from Tshopo in the Democratic Republic of Congo. It is used as food, as medicine, for recreational aspect, and for myth and belief. The mythical and religious purposes might confirm the hallucinogenic effects of fungi on people. However, it makes belief that spiritual interaction may exist between wild fungi and people. Saprotrophic fungi, including many wood-decaying taxa, are the most consumed and appreciated because of their taste. Some mushroom pickers deliberately cut down host trees to promote mushroom development, eventually promoting or boosting forest degradation. For sustainable use of natural forests and to mitigate the harvest-related pressure on natural habitats, the cultivation of the most useful and appreciated saprotrophic fungi (value MCSI above 1) such as *Auricularia cornea*, *Marasmius buzungolo*, *Pleurotus tuber-regium*, *Lentinus squarrosulus*, and *Schizophyllym commune* is proposed. However, the latter species should be worth discarded from culturing as it is known to cause the severe “Schizophyllum disease” in both healthy and immunodeficient people [51, 52].

## Abbreviations

BUI: Belief and myth use index
CA: Correspondence analysis
CERUKI: Centre de Recherches Universitaires du Kivu
CS: Cultural significance
DR Congo: Democratic Republic of the Congo
EICS: Ethnic index of cultural significance
MCSI: Mushrooms’ cultural significance index
MEI: Mushrooms’ edibility index
MUI: Medicinal use index
NT: Number of types of uses
NTFP: Non-timber forest product
NUC: Number of use categories
RI: Relative importance
RU: Reported uses
RUI: Recreational use index
TAI: Taste appreciation index
